# Supplementary material for: Responsiveness of monopodal postural stability tests in recreational athletes
Source: PeerJ. 2024 Jan 11;12:e16765. doi: 10.7717/peerj.16765 (PMC10788087; doi:10.7717/peerj.16765)
Supplement: Supplemental Information 2 [file peerj-12-16765-s002.docx]

VARIABLES:

SEX: 1- MALE 2-FEMALE

DOM: DOMINANT

NDOM: NO DOMINANT

STABILOMETRY VARIABLES:

- AREA
- VELOCITY
- XMEAN
- YMEAN

YBT: Y-balance Test

DOMA: DOMINANT anterior

DOMPM PM: DOMINANT posteromedial

DOMPL: DOMINANT posterolateral

ET: Emery balance test
