# Supplementary material for: Responsiveness of monopodal postural stability tests in recreational athletes
Source: PeerJ. 2024 Jan 11;12:e16765. doi: 10.7717/peerj.16765 (PMC10788087; doi:10.7717/peerj.16765)
Supplement: Supplemental Information 3 [file peerj-12-16765-s003.pdf]

Supplementary Appendix 1. Flow chart of the study participants.

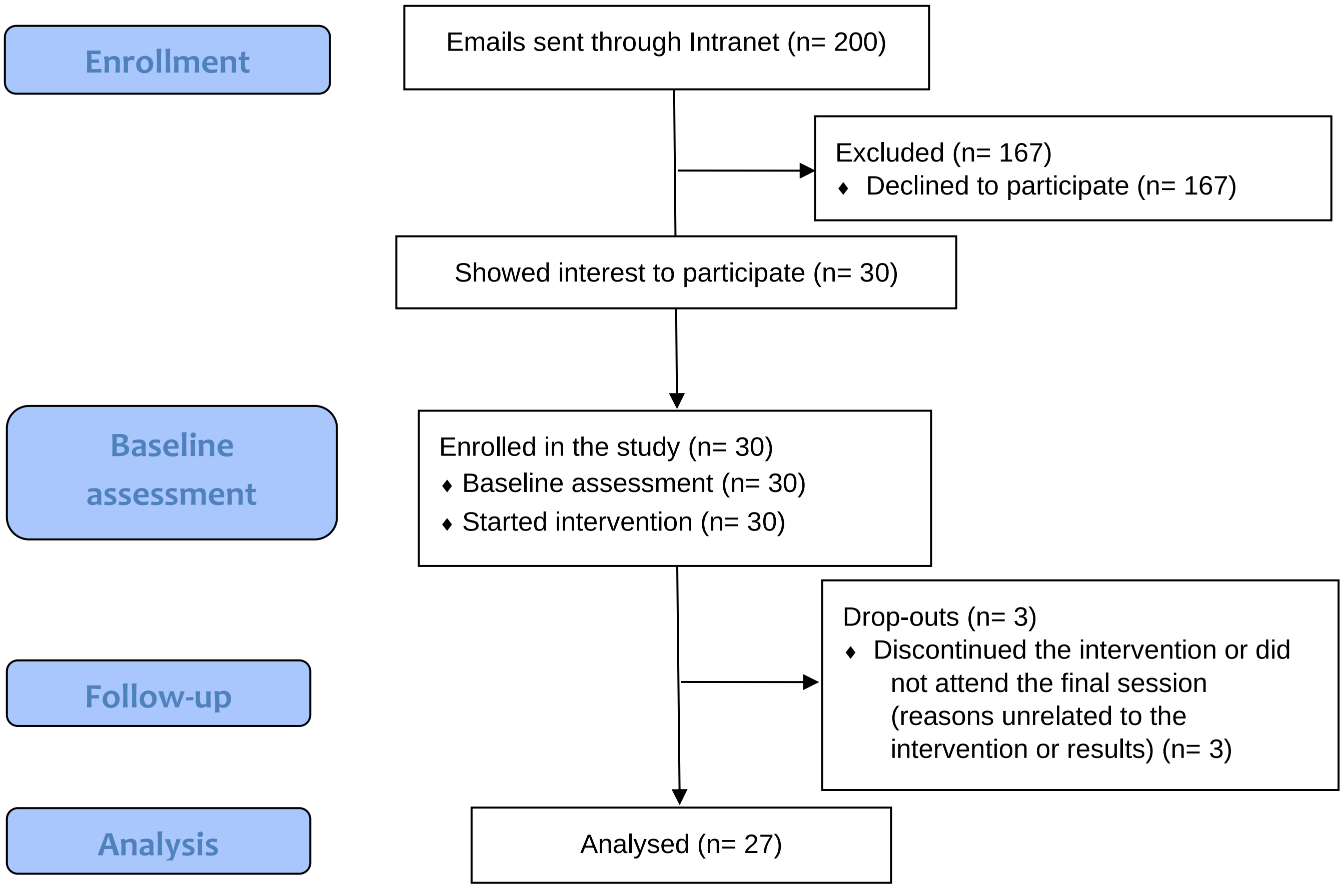

Supplementary Appendix 2. Individual values for all participants.

Supplementary Appendix 2.1 Individual values for Emery Balance Test (dominant lower limb).

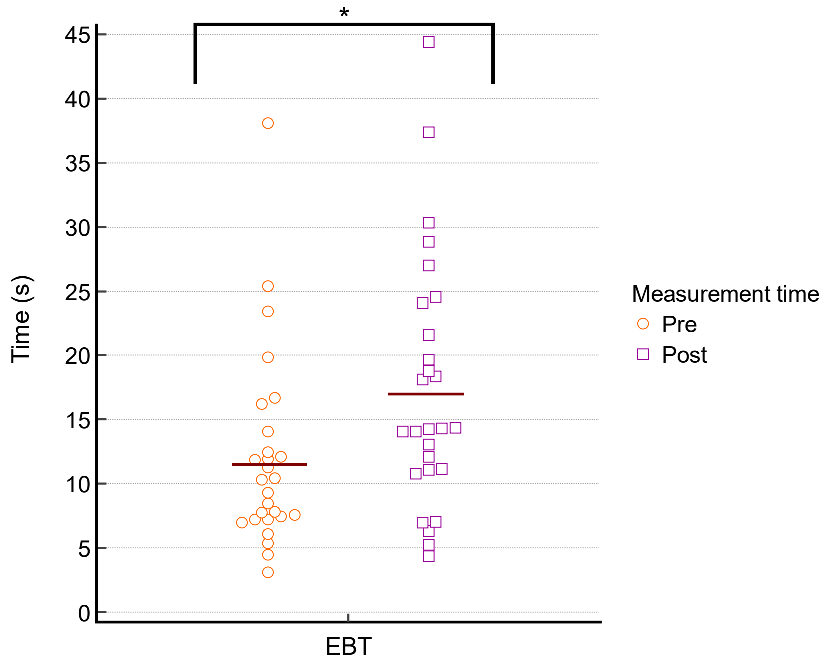

The connector with the asterisk (*) represents the existence of statistically significant differences between the pre- and post-intervention measurement. The horizontal line of each group of circles/squares represents the mean value. EBT: Emery balance test

Supplementary Appendix 2.2 Individual values for modified Star Excursion Balance Test (dominant lower limb).

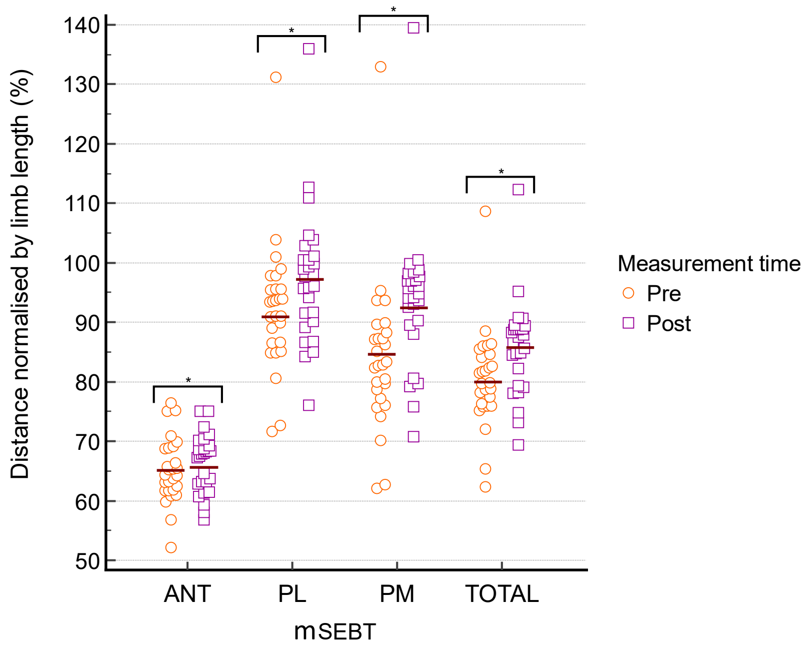

The connector with the asterisk (*) represents the existence of statistically significant differences between the pre- and post-intervention measurement. The horizontal line of each group of circles/squares represents the mean value. mSEBT: modified Star Excursion Balance Test; ANT: anterior; PM: posteromedial; PL: posterolateral

Supplementary Appendix 2.3 Individual values for area values of Stabilometry (dominant lower limb).

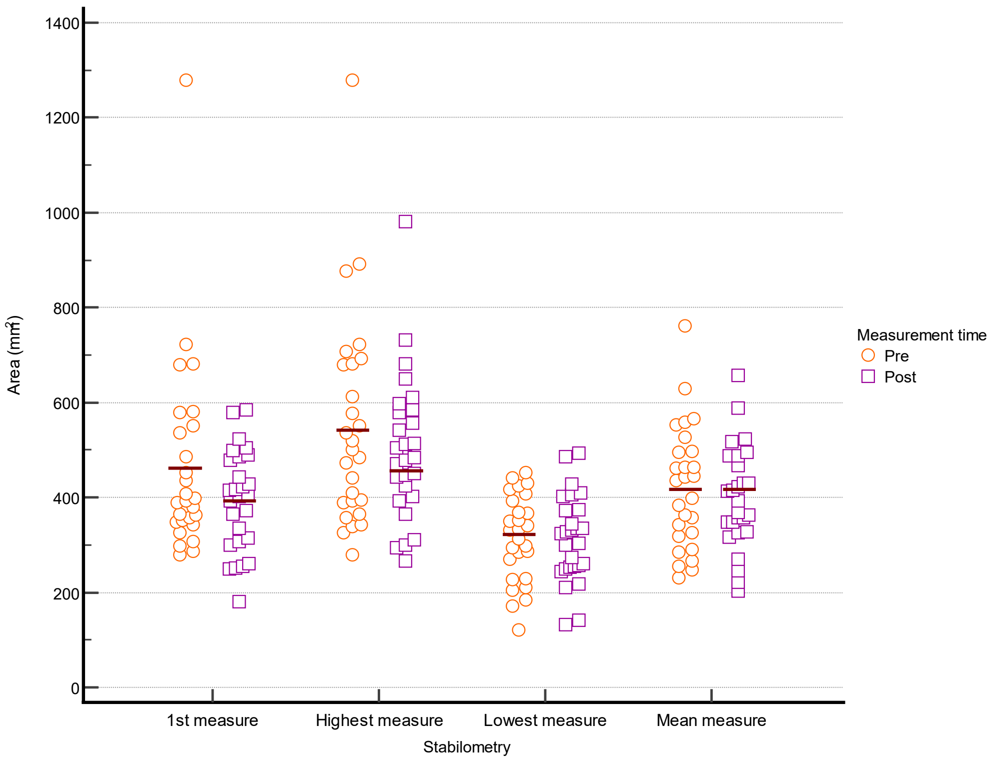

The horizontal line of each group of circles/squares represents the mean value.

Supplementary Appendix 2.4 Individual values for velocity values of Stabilometry (dominant lower limb).

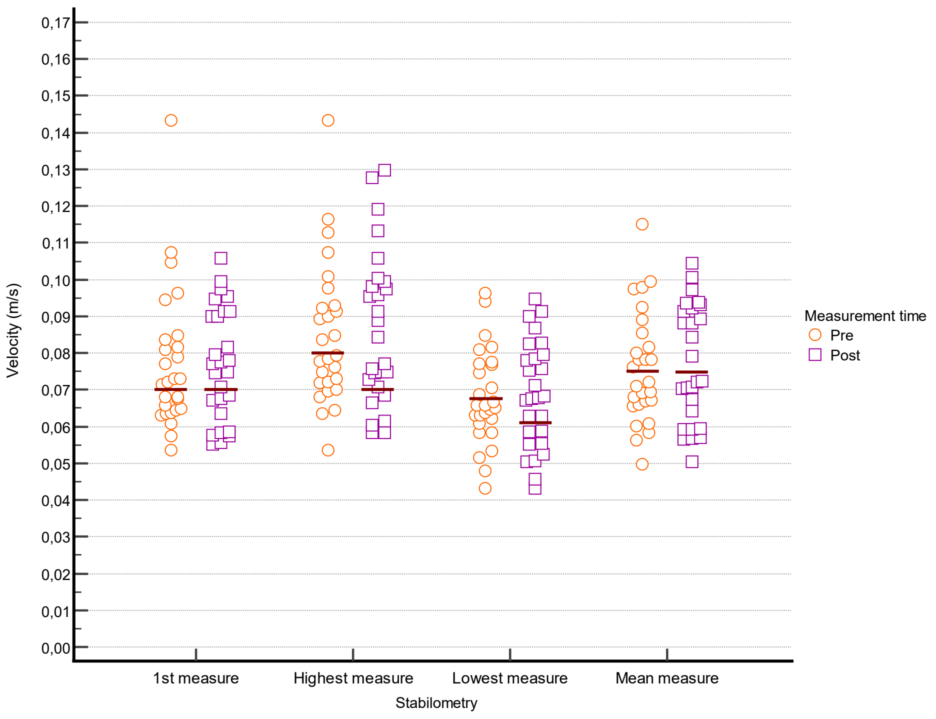

The horizontal line of each group of circles/squares represents the mean value.

Supplementary Appendix 2.5 Individual values for X mean values of Stabilometry (dominant lower limb).

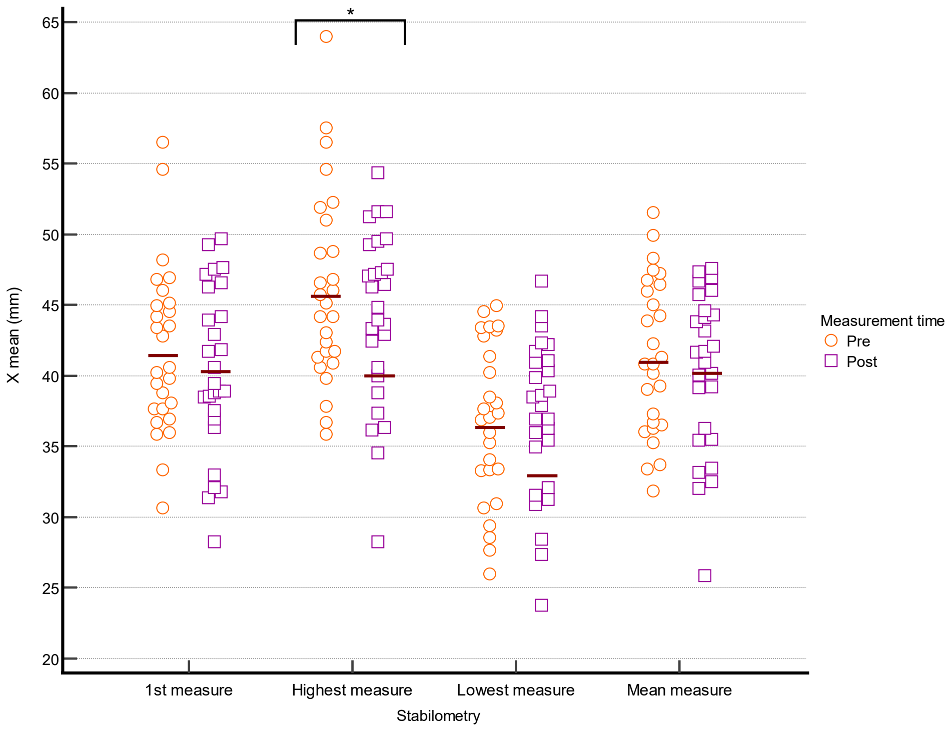

The connector with the asterisk (*) represents the existence of statistically significant differences between the pre- and post-intervention measurement. The horizontal line of each group of circles/squares represents the mean value.

Supplementary Appendix 2.6 Individual values for Y mean values of Stabilometry (dominant lower limb).

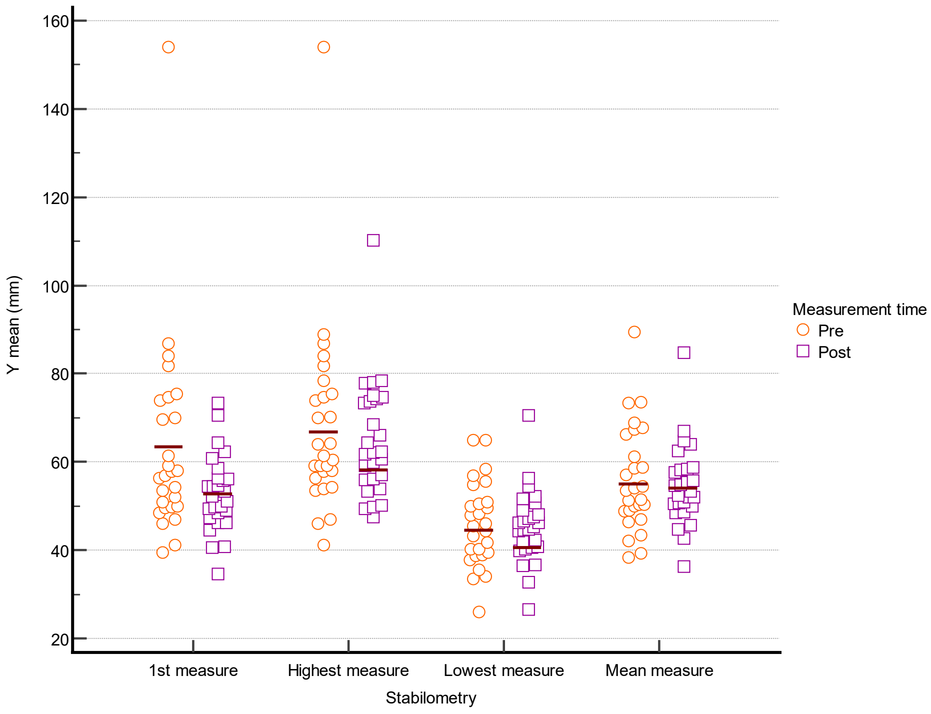

The horizontal line of each group of circles/squares represents the mean value.
